# Simultaneous Versus Sequential IMRT Boost in the Era of Treatment De-Escalation of Head and Neck Cancers

**DOI:** 10.3390/cancers18091339

**Published:** 2026-04-23

**Authors:** Emily H. Evani, Esther Yu, Parisa Shamsesfandabadi, David M. Brizel, Jared R. Robbins

**Affiliations:** Department of Radiation Oncology, Duke University, 20 Duke Medicine Circle, Durham, NC 27710, USA; emily.m.harris@duke.edu (E.H.E.);

**Keywords:** head and neck cancer, IMRT, sequential boost, simultaneous integrated boost

## Abstract

Radiation therapy is a central component of the definitive and postoperative management for head and neck cancers (HNC), with intensity-modulated radiation therapy (IMRT) and volumetric modulated arc therapy (VMAT) now standard. Within these techniques, two principal boost strategies are used: simultaneous integrated boost (SIB) and sequential boost (SEQ). Although both are guideline-supported, they differ in planning logistics, treatment delivery, potential radiobiologic effects, adaptability to anatomic change, and potential toxicity profiles. In this narrative review, we summarize the key technical, dosimetric, and radiobiologic differences between SIB and SEQ and synthesize the available comparative clinical data, with a focus on their roles in contemporary dose de-escalation strategies. SIB allows for differential dosing within a single plan and potentially shorter overall treatment time but typically delivers higher biologically effective doses (BED) to elective nodal regions. SEQ requires two plans but offers greater flexibility for adaptive replanning, facilitates a lower BED to elective nodal volumes, and may allow for partial normal tissue recovery during the boost phase. Comparative studies, including retrospective series, randomized trials, and a meta-analysis, have not demonstrated consistent differences between SIB and SEQ in survival or local control, with mixed findings regarding toxicity. In the context of de-escalation, multiple prospective studies have successfully used SEQ to reduce elective nodal dose with low rates of elective nodal failure, while recent data suggest that SIB-based elective dose reduction may also be feasible in select settings. Overall, both SIB and SEQ are effective boost strategies in HNC radiotherapy. While practice is often driven by institutional workflow and clinician preference, emerging evidence suggests potential advantages of SEQ for elective nodal dose de-escalation. Further prospective studies are needed to better define the relative impacts of SIB and SEQ on toxicity and tumor control.

## Simple Summary

This narrative compares IMRT/VMAT boost strategies used in the treatment of head and neck cancers: simultaneous integrated boost (SIB) and sequential boost (SEQ). Both techniques are widely used and achieve comparable outcomes in tumor control and survival. SIB delivers differential doses to tumor and elective regions concurrently within a single plan, enabling simpler workflows, excellent conformality, and potential for shortening treatment duration. In contrast, SEQ uses a two-phase approach which allows for sparing of normal tissues during the boost and can provide for purposely biologically effective dose (BED) reduction to elective nodal regions. Clinical studies, including randomized trials and meta-analyses, show no consistent differences in efficacy, though toxicity findings are mixed. While most emerging evidence supports SEQ in de-escalation strategies, a recent study demonstrated that similarly low elective BED can be safely achieved with SIB. Overall, both techniques remain appropriate, with selection guided by clinical context, workflow, and evolving evidence.

## 1. Introduction

Head and neck cancers account for about 4% of diagnosed malignancies each year in the United States, with approximately 71,000 people diagnosed with a head and neck cancer in 2024 [1]. This group of malignancies consists of cancers of the oral cavity, oropharynx, nasopharynx, paranasal sinuses, salivary glands, and larynx. Risk factors for head and neck cancers include tobacco use, alcohol use, and HPV exposure [2]. Radiation therapy (RT) is a key component of the multidisciplinary approach to the treatment for many head and neck cancers.

When radiation therapy is utilized for the treatment of head and neck cancers, it is typically delivered through a high dose of radiation to the tumor and involved or pathologically enlarged lymph nodes, and a lower dose to an elective volume of lymph node levels at risk for subclinical disease. This high dose delivered to gross disease is commonly referred to as a “boost”.

As radiation techniques have progressed, intensity modulated radiation therapy (IMRT) and volumetric modulated arc therapy (VMAT) have increasingly become the modalities of choice for the treatment of head and neck cancers. IMRT has been shown to reduce treatment-related toxicity while improving disease coverage in this patient population [3,4,5]. With IMRT, a radiation boost to high-risk volumes can be achieved using one of two techniques: a simultaneous integrated boost (SIB) or a sequential boost (SEQ). With a SIB, the high-risk boost volume receives the total prescribed dose while the elective treatment volume is concurrently treated, with risk-based differences in the dose per fraction to each volume (i.e., boost volume receives 2 Gy per fraction with elective coverage receiving as low as 1.5 Gy per fraction). In comparison, when utilizing an SEQ, the entire treatment volume receives a prescribed dose of radiation, which is followed by a treatment with an additional dose of radiation only to the boost volumes. Both SIB and SEQ are accepted treatment techniques within the head and neck radiation community [6], and each comes with its own set of advantages and disadvantages, as detailed in Table 1.

This review will focus on the utility, logistics, and literature surrounding SIB and SEQ in radiation treatment, with a special focus on the advantages and disadvantages of each technique in the current era of head and neck treatment de-escalation.

## 2. Sequential Boost (SEQ)

A sequential boost (SEQ) is achieved by the delivery of a prescribed dose to the entire treatment volume followed by a boost to a high-risk volume by generating two separate treatment plans. SEQ techniques generally deliver a uniform fraction size to a larger volume followed by a cone-down boost, resulting in more homogeneous fractionation but a longer effective treatment time for the high-risk region. An example of this technique in oropharynx cancer would be to treat the low-risk volume to 50 Gy in 25 fractions, followed by treatment of the high-risk volume with an additional 20 Gy in 10 fractions to a total dose of 70 Gy.

A key consideration during head and neck radiotherapy is the dynamic nature of treatment volumes over the course of therapy. Changes may occur for multiple reasons, including treatment-related weight loss, edema, and tumor response. Patients frequently experience weight loss due to the acute toxicities of radiation therapy or chemoradiation, such as mucositis with odynophagia, xerostomia, and dysgeusia. This weight loss may ultimately lead to treatment volumes, which become less conformal over time due to significant decreases in fat and muscle mass throughout the course of therapy. In parallel, gross tumor volume (GTV) can decrease dramatically throughout the course of treatment. Each of these scenarios may necessitate re-simulation and re-planning during a patient’s treatment course to maintain target coverage and spare organs-at-risk. An advantage of a SEQ is that when a new boost plan is required, it can be tailored to the current patient and target volumes rather than replanning all treatment volumes, as would be required with SIB. This allows one to focus solely on the replanning of the boost volume, which may save time and resources from a planning perspective.

On the other hand, an SEQ requires more legwork in the upfront planning process as two separate plans are generated for treatment: the initial plan for the entire treatment volume and an additional plan for the boost volume. In comparison, a single plan is generated using an SIB, decreasing the workload for radiation planners.

One disadvantage of SEQ is the possible longer overall treatment time when compared to SIB, which can be especially challenging for patients with a poor performance status. For example, in the post-operative setting (i.e., close or positive margins, areas of extranodal extension), an additional boost to high-risk areas or even residual disease can be delivered with either technique, but with SEQ, the treatment would be delivered in 33 treatments, while with SIB the area of concern could receive 2.2 Gy per fraction, allowing for treatment completion in only 30 fractions. Likewise, in the definitive setting, a common SIB fractionation scheme is 69.96 Gy in 33 fractions, which is possible by giving 2.12 Gy per fraction to the high-risk areas and a lower dose per fraction to the intermediate- and low-dose volumes. When SEQ is used in the definitive setting, a common scheme would be 70 Gy in 35 fractions, with a slightly longer treatment time when compared to the SIB scheme. The longer course of treatment using SEQ may increase the risk of treatment interruptions. These distinctions have implications for tumor repopulation, as the prolongation of overall treatment time and unplanned interruptions may reduce tumor control probability, whereas a modest fraction size escalation may increase biologically effective dose (BED) to the gross disease.

A potential benefit of the SEQ technique is that the elective volume is spared from receiving treatment during the boost portion of the treatment. This reduces the incidental dose of radiation to non-target normal tissues in the elective volume such as skin and, in some instances, hypopharyngeal mucosa, which can allow some healing to begin even as the boost volume is delivered. This expedition of the healing process of normal tissues in the elective volume may help patients progress through their treatment courses with less discomfort.

Radiobiologic considerations differ between SIB and SEQ approaches, primarily due to variations in dose per fraction and overall treatment time. Using an SEQ allows for lower biological equivalent dose (BED) coverage of elective nodal areas. There is a lower limit to the fractional dose that can be delivered to elective nodal areas while ensuring that treatment remains effective. With SEQ there have been reports of elective doses as low as 30 Gy, which would not be possible with an SIB technique. The ability to reduce the BED to the elective nodal volume and nearby normal tissues is a unique advantage of the SEQ technique, especially in very responsive tumors with excellent prognosis, such as HPV+ oropharyngeal cancer, where long-term toxicity reduction is essential.

## 3. Simultaneous Integrated Boost (SIB)

In SIB schedules, the high-dose target volume typically receives a slightly higher dose per fraction while elective regions receive a lower dose per fraction during the same treatment course, creating differential biologic dosing within a single plan. The ability to deliver a variety of doses simultaneously to different volumes of tissues without increasing the overall treatment time is a notable advantage of SIB when compared to SEQ. An example of this is the dosing schema utilized by RTOG 1016, with 70 Gy delivered to high-risk volumes, 59.5 Gy delivered to intermediate-risk volumes, and 52.5 Gy delivered to low-risk volumes [7].

A potential advantage of SIB is the increased dose per fraction delivered to the GTV. This has been hypothesized but not proven to enhance tumor control due to an increase in the biologically effective dose (BED). One example of this technique would be to treat a high-risk PTV to approximately 70 at 2.12 Gy per fraction in 33 fractions. Additionally, many studies evaluating definitive hypofractionated radiation rely on an SIB technique to deliver a high dose per fraction to the gross disease while treating the elective nodal areas with more standard fractionation. For example, the HYPNO trial delivered 55 Gy in 20 fractions to gross disease (2.75 Gy/fraction) and 40 Gy in 20 fractions to elective nodes (2 Gy/fraction) [8]. Furthermore, delivering a higher dose to the tumor volume early in treatment with an SIB technique may reduce the risk of tumor cell repopulation.

As previously discussed, a fundamental advantage of SIB is the efficiency of the upfront treatment planning process when compared to SEQ. This is especially beneficial at high-volume cancer centers.

## 4. Comparing SEQ and SIB Dosimetry

**Figure 1:**
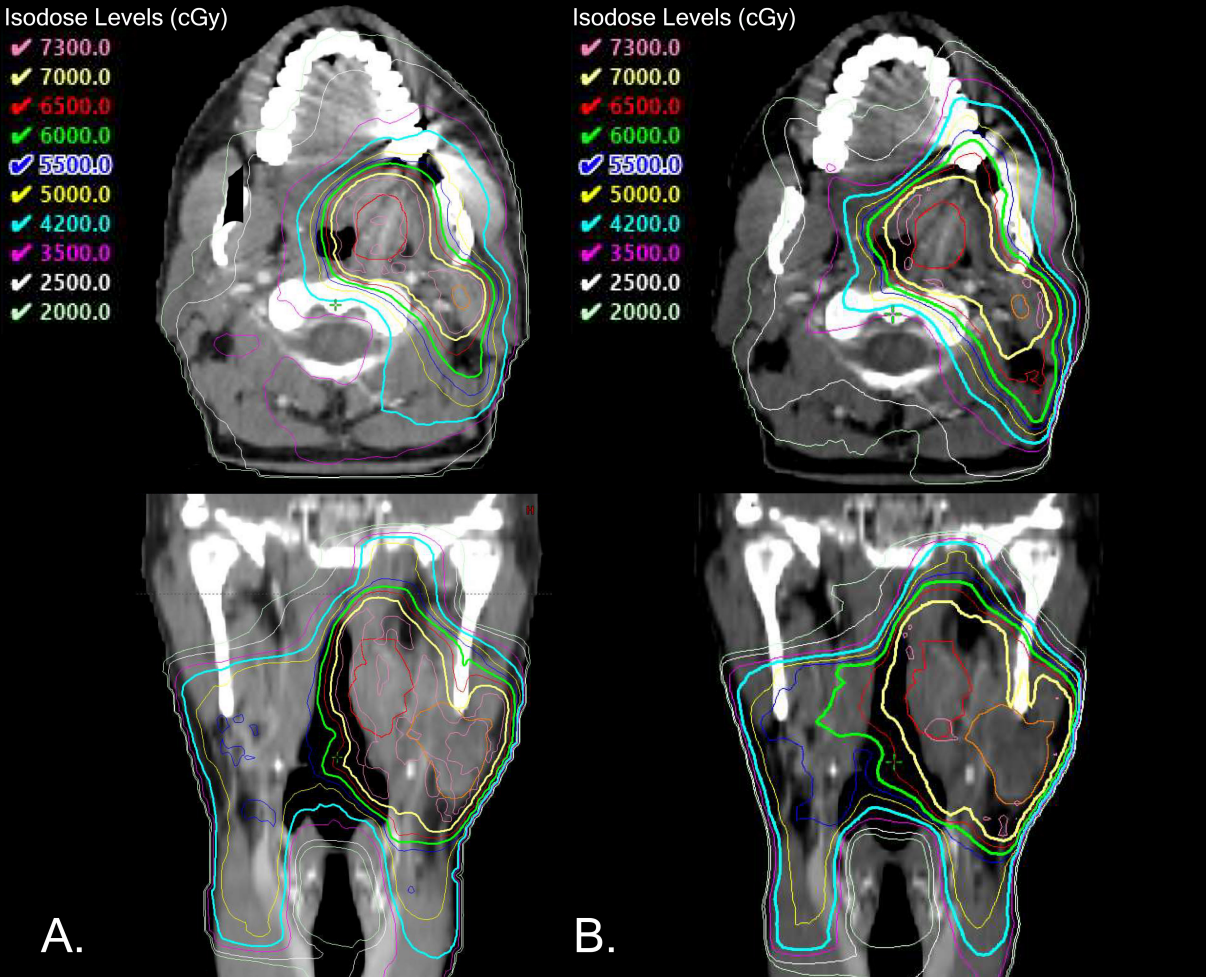

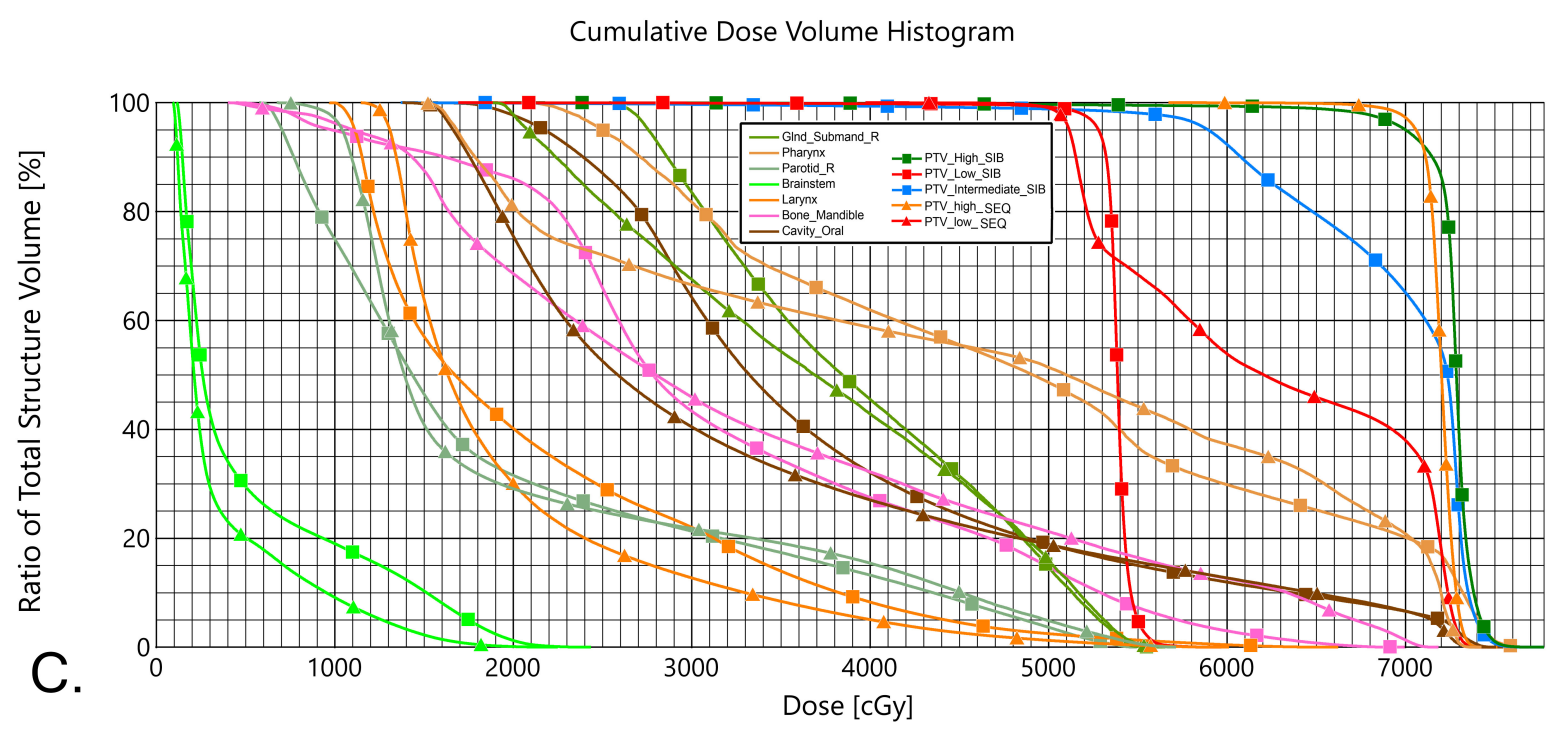
Comparison of simultaneous integrated boost (SIB) and sequential boost (SEQ) dosimetric outcomes for a representative HPV-positive oropharyngeal cancer case. (**A**) SIB treatment plan. (**B**) SEQ treatment plan. (**C**) Dose–volume histogram (DVH), with SIB curves denoted by squares and SEQ curves denoted by triangles. (Source: original creation of study team to illustrate differences between delivery techniques.)

When comparing treatment plans utilizing SEQ versus SIB techniques, SIB plans tend to be more conformal, providing the ability to better control dose distributions (Figure 1). This can lead to sharper dose fall-off and protection of normal structures but may necessitate the inclusion of an intermediate dose volume to reduce the risk of marginal misses adjacent to high-risk/boost targets. In comparison, with SEQ delivery intermediate doses are created from the penumbra of the boost dose and by virtue of beams traversing normal and elective volumes to reach the boost targets. SIB requires an intermediate dose volume to be included in radiation planning as this volume is not an inherent by-product of treatment, as with SEQ. Additionally, monitoring the daily accuracy of the treatment setup and conformality of the treatment volumes with daily image guidance is especially important with SIB plans to assess the need for re-simulation and/or re-planning due to weight loss and/or treatment response.

## 5. SEQ Versus SIB: What Is Known and What Is Debated

Practice patterns regarding SEQ versus SIB techniques vary across institutions as there is limited data to support one technique over the other regarding survival or toxicity outcomes.

In a retrospective review of 54 patients with oropharynx cancer treated with definitive radiation therapy using VMAT with either SIB or SEQ, no significant differences were observed in overall survival (OS), disease-free survival (DFS), or local control (LC) [9]. However, the SEQ cohort experienced higher rates of late toxicity when compared to the SIB cohort, including xerostomia (42% versus 24.2%) and dysphagia (33.3% versus 15.1%). Conversely, a separate retrospective analysis of 209 patients with locally advanced head and neck cancer treated with IMRT found that SIB was associated with higher rates of grade 3 or 4 dysphagia (82% versus 55%) and dermatitis (78% versus 58%), but found no differences in late toxicities or survival outcomes [10]. A third retrospective study of 182 patients with oral cavity cancer undergoing post-operative radiation treatment found higher rates of mortality with SIB-IMRT when compared to SEQ-IMRT (36.8% versus 20.0%), as well as higher rates of primary recurrences (26.3% versus 10.0%) and marginal failure rates (26.7% versus 16.7%) [11].

Broader pooled and population-based analyses have generally not demonstrated meaningful oncologic differences between the two approaches. A meta-analysis including approximately 1000 patients with head and neck cancers treated with IMRT across seven studies found no difference in OS, DFS, LC, or acute toxicities when comparing SIB and SEQ [12]. Likewise, a population-based propensity score-matched analysis from Taiwan evaluating 200 patients with oropharynx cancer or hypopharynx cancer treated with definitive concurrent chemoradiation found no significant difference in survival outcomes between patients treated with SIB and SEQ IMRT [13].

Prospective studies have similarly shown inconsistent toxicity findings, while largely failing to demonstrate clear differences in tumor outcomes. A prospective randomized controlled trial of 110 patients with locally advanced head and neck cancers compared acute toxicities and response rates found significantly higher rates of grade 3 acute dysphagia with SIB when compared to SEQ (72% versus 41.2%), but found no difference in response rates at 3 months [14]. A second prospective trial of 66 patients with locally advanced head and neck cancer showed higher rates of acute grade 3 dysphagia with SIB-IMRT when compared to SEQ-IMRT (45.5% vs. 24.2%), but no difference in OS or PFS at 4 years [15]. Similarly, a third prospective study included 60 patients with head and neck squamous cell carcinoma and found higher rates of grade 3 acute mucositis with SIB when compared to SEQ treatment (43.3% versus 10%), and there was no significant difference in OS between the two groups at 2 years [16]. In contrast, a prospective study of 209 patients with nasopharynx cancer treated with IMRT randomized to SEQ or SIB found no differences in grade 3–4 acute toxicities or in progression-free survival (PFS) and OS at 3 years [17]. Finally, a prospective comparison of ototoxicity outcomes in head and neck cancer patients treated with SEQ or SIB VMAT showed no significant differences in sensorineural hearing loss or survival between the two approaches [18]. Given the lack of substantial data to support one technique over the other, the choice between SIB and SEQ when treating patients with head and neck cancers is often attributed to provider preferences. Ultimately, both SIB and SEQ are techniques that are supported by NCCN guidelines for head and neck cancers.

## 6. Dose De-Escalation in HNC and Boost Technique

Human papilloma virus (HPV) expressing head and neck tumors have been shown to be especially responsive to treatment with chemoradiation [19]. Importantly, HPV-positive cancers comprise the majority of oropharynx cancer diagnoses in the United States today [20]. This finding has led to various studies exploring methods to de-intensify treatment with the goal of decreasing the toxicity of curative treatment while maintaining the high probability of cure. In radiation oncology, efforts to improve the therapeutic profile of curative radiation have focused on de-escalating the total dose of radiation to the primary tumor and elective nodal volume, as well as limiting the volume of irradiated tissue. In particular, the focus on the minimum dose necessary to sterilize the lymphatics at risk has further brought into question the best approach to treatment planning regarding SIB versus SEQ.

Tsai et al. at MSKCC conducted a retrospective cohort study evaluating the de-escalation of radiation dose to the elective nodal and sub-clinical volumes in patients with HPV-positive oropharynx cancer [21]. Elective dose was reduced to 30 Gy in 15 fractions, which was followed by an SEQ of 40 Gy in 20 fractions to gross disease to a total of 70 Gy. LC, PFS, and OS at 2 years were 97%, 88%, and 95.1%, respectively.

A single-arm phase 2 study by Maguire et al. from Coastal Carolina showed the potential for de-escalation of the elective nodal radiation dose in locoregionally advanced head and neck cancer [22]. Patients were treated with 36 Gy in 18 fractions to high-risk and elective volumes, followed by an SEQ of 34 Gy in 17 fractions to the high-risk volume. The study reported no elective nodal failures and an actuarial 3-year survival rate of 96% for HPV-positive patients.

A prospective, dual-center, phase 2 study evaluated the feasibility of dose de-escalation to the elective nodal volume in oropharynx and larynx cancers. This trial uniquely used a combination of SIB and SEQ techniques. During the initial phase of treatment, an SEQ was used to treat all gross disease, suspicious nodes, and elective volumes with a uniform dose of 40 Gy in 20 fractions. During the second phase of treatment an SIB was used to simultaneously boost gross disease to an additional 30 Gy (2 Gy/fraction), while suspicious nodes received an additional 24 Gy (1.6 Gy/fraction) in 15 fractions for a total of 70 Gy and 64 Gy, respectively. There were no solitary elective nodal recurrences at a median follow-up of 24.7 months [23].

A recent study, JCOG 1912, was presented at the European Society of Medical Oncology in 2025, which evaluated SEQ IMRT techniques as a means of reducing the radiation dose to the elective nodal volume in HPV-negative HNC [24]. This phase 3 randomized trial compared a reduced elective nodal volume radiation dose of 40 Gy in 20 fractions delivered via SEQ versus a standard dose of 56 Gy in 35 fractions delivered via SIB in patients with p16-negative locally advanced squamous cell carcinoma of the head and neck. All patients received a definitive dose of 70 Gy to gross disease with concurrent cisplatin. The trial was terminated early due to futility, with a 2-year time to treatment failure rate of 72.5% in the 56 Gy SEQ arm compared to 57.7% in the 40 Gy SEQ arm. Though the trial failed to demonstrate non-inferiority of the dose in the de-escalated 40 Gy SEQ arm, there, importantly, was no difference seen in 2-year time to treatment failure between the two treatment arms among patients who were clinically node-negative (~71% in both arms) or differences in the recurrences in the low-risk elective nodal volumes.

As prior discussed, one potential advantage of SEQ is the ability to de-escalate dose to elective volumes without decreasing the dose per fraction to a level that may not be effective. While prior elective nodal de-escalation trials utilized the SEQ technique, the UPGRADE-RT trial provides evidence that dose-reduction in the elective volume in HNC radiation using an SIB may be a viable option. This study challenges the notion that the coverage of elective nodal volumes with a low dose per fraction may be ineffective. This multicenter randomized trial included newly diagnosed patients with cT2-4N0-2M0 HNC who were treated to 68 Gy in 34 fractions with an elective nodal irradiation dose and randomly assigned to a dose reduction of 43 Gy in 34 fractions (1.26 Gy/fraction) or a control dose of 50 Gy in 34 fractions (1.47 Gy/fraction). The 2-year recurrence rates were 4.9% and 4.3% in the dose reduction and control groups, respectively, and the toxicity was lower in the dose-reduced arm [25]. This suggests that lowering the dose per fraction to elective volumes (about 1.3 Gy/fraction in this case) may be sufficient for disease control in low-risk areas.

On the other hand, NRG-HN005, which utilized an SIB technique, failed to demonstrate non-inferiority of dose de-escalation. This phase 2 randomized trial evaluated radiation de-escalation with systemic therapy in patients with low-risk, HPV-positive, non-smoking-associated oropharynx cancer [26]. The trial randomized patients to receive a standard dose of radiation to 70 Gy in 35 fractions with cisplatin (6 fractions per week), a reduced dose of radiation to 60 Gy and 30 fractions with cisplatin (5 fractions per week), or a reduced dose of radiation to 60 Gy in 30 fractions with nivolumab (6 fractions per week). Uninvolved nodes were treated electively using an SIB to 52.5 Gy and 48 Gy in the 70 Gy and 60 Gy arms, respectively. At interim futility analysis, the radiation dose de-escalation arms failed to demonstrate non-inferiority compared to standard radiation to 70 Gy with concurrent cisplatin, and the trial did not proceed to phase 3. Investigators of this trial noted that this failure of the de-escalation arms to show non-inferiority is at least partly due to the high survival rates of patients treated with the standard treatment regimen (98% PFS at 2 years). We await further information regarding the patterns of failure, particularly in the elective nodal areas and gross disease in the de-escalated arms.

Table 2 shows a summary of the elective nodal doses used in various head and neck clinical trials, showing the difference in BED3 and BED10.

## 7. Emerging Data

As evidence continues to emerge regarding the efficacy and safety of dose de-escalation for head and neck cancers, ongoing studies aim to home in on who may benefit most from this approach.

The Re-ACT Study is a phase II clinical trial evaluating the efficacy of risk-adapted radiation in HPV-positive oropharynx cancer utilizing NavDx, a blood test which detects circulating tumor DNA from HPV-driven cancers. Based on NavDx results, patients with a predetermined level of response to initial treatment received a de-escalated treatment, while those who failed to achieve this response received a standard course of radiation to 70 Gy in 35 fractions. In this trial, they allowed for de-escalation using either an SIB plan of 60 Gy to gross disease and 48 Gy to elective nodal volumes in 30 fractions or an SEQ plan of 30 Gy in 15 fractions to elective nodes and gross disease followed by a boost to 60 Gy to gross disease [30]. The primary endpoint of this study is PFS at 2 years. The initial results were presented at ASCO 2025 and reported a 2-year PFS of 91% for intermediate-risk de-escalated patients, which was similar to the 2-year PFS of 92% for low-risk de-escalated patients [30].

A prospective single-arm non-inferiority trial conducted at the Memorial Sloan Kettering Cancer Center aimed to evaluate the utility of using a ^18^F-flourmisonidazole (F-MISO) PET/CT to identify patients who could be candidates for de-escalated radiation. F-MISO can be used to measure hypoxia, and in this study of HPV-associated oropharynx cancers, patients who had their primary tumor removed with a negative margin were assigned radiation dose based on the results of the baseline and mid-treatment F-MISO PET/CT scans. Patients with either no base-line hypoxia or those who were hypoxia-negative on the interim F-MISO PET/CT were treated with 30 Gy/15 fractions, while patients whose tumors remained hypoxic received 30 Gy/15 fractions to elective nodes, followed by an SEQ of 40 Gy/20 fractions for a total dose of 70 Gy/35 fractions [29]. This study demonstrated non-inferiority in 2-year locoregional control, with similar PFS (94% vs. 96%) and OS (100% vs. 96%) for the de-escalated and the standard cohorts, while the grade 3–4 acute and late toxicity was higher in the 70-Gy cohort.

Two prospective studies conducted at Duke University and the University of Michigan are evaluating the feasibility of using interim FDG-PET/CT to guide dose de-escalation for patients with HPV-positive oropharynx cancer. While both protocols call for dose de-escalation for patients who achieve appropriate reductions in FDG avidity after 10–12 fractions, they use different boost techniques and doses in the de-escalated arms. The Duke protocol utilizes SEQ, where patients that do not qualify for de-escalation receive a standard treatment of 44 Gy to the elective volume and 70 Gy to gross disease, while good responders receive a reduced dose of 36 Gy to the elective volume and 60 Gy to gross disease. In comparison, the Michigan study uses an SIB technique with standard patients receiving 56 Gy to the elective volume and 70 Gy to gross disease in 35 fractions, while good responders receive 43.2 Gy to the elective volume and 54 Gy to gross disease in 27 fractions. The primary endpoints are 2-year PFS and locoregional relapse for the Duke and Michigan studies, respectively [27,28]. While the Duke trial is ongoing, the Michigan authors have reported a 43% rate of de-escalation in enrolled patients, and a 24-month locoregional relapse rate for the entire cohort of 7.8%. While we await the outcomes of the Duke trial, the Michigan results are encouraging and suggest the feasibility of response-adapted treatment de-escalation in HPV-positive HNC.

As demonstrated above, a growing number of de-escalation trials are using sequential boost techniques as a method to decrease the dose to elective nodal volumes. To date, these studies have reported low rates of elective nodal failure, which may indicate that a low BED is sufficient to eradicate microscopic disease, and higher BEDs may ultimately be overtreating these elective nodal areas. Evidence to support the potential overtreatment of elective nodal volumes includes a systematic reduction in the recommendations for elective nodal coverage in nasopharynx cancer. The newest guidelines suggest specific instances where several elective nodal levels (Ib, IV, and medial retropharyngeal lymph nodes) may be dose-reduced or even eliminated [31]. Evidence-based elective nodal coverage and appropriate elective nodal irradiation dose can substantially reduce the risk of toxicity. As such, efforts to further evaluate both the dose and volume of elective nodal irradiation are warranted to further personalize treatment and limit toxicity while enhancing efficacy. Radiation boost technique will likely play an important role in this effort and will be an important element of future innovative trials and study concepts.

## 8. Conclusions

Both SIB and SEQ are boost techniques that are frequently used by radiation oncologists when treating HNC. In contemporary practice, selection is often influenced by institutional workflow and clinician preference, but increasingly also by treatment intent and evolving trial design. With emerging data regarding dose de-escalation in HNC, many trials are adopting the SEQ technique due to the ability to deliver a lower BED to elective nodal areas without significantly reducing the daily dose per fraction too drastically, which would be required with an SIB technique. Though some concerns about the de-escalation of treatment to low-risk elective nodes with a very low dose per fraction using an SIB technique may be somewhat quieted by the recently published UPGRADE-RT trial (which utilized <1.5 Gy/fraction and <1.3 Gy/fraction in their treatment arms), additional studies and longer follow-up are needed before making any firm conclusions. While there is currently no evidence to definitively support one technique over the other for most head and neck treatment plans, SEQ may be advantageous for multiple logistical, anatomical, and radiobiological reasons in the setting of the de-escalation of treatment to elective nodal regions.

## 9. Future Directions

As evidence in this space continues to build, the presence (or lack thereof) of any significant difference in disease outcomes when using SIB versus SEQ techniques remains to be determined. Additionally, there is little consistent data regarding differences in acute and late toxicities between these two techniques. Future studies exploring these differences are warranted.

## Figures and Tables

**Table 1:** Simultaneous Integrated Boost vs. Sequential Boost.

Category	Simultaneous Integrated Boost	Sequential Boost
Treatment Time	Shorter overall treatment duration	Longer overall treatment duration
Dose Delivery	Boost delivered concurrently throughout treatment.	Boost delivered in a separate phase after initial treatment.
Planning Simplicity	Single treatment plan	Requires separate boost planning
Adaptability	Less opportunity for mid-treatment adaptation.	Allows for re-planning based on anatomical changes before boost phase.
Fractionation	Uses different doses per fraction across target volumes.	Uniform dose per fraction across all phases.
Tumor Control Potential	Potential to deliver more than 2 Gy to the tumor, which may reduce risk of repopulation.	Potentially increased risk of repopulation due to delayed boost.
Treatment Interruptions	Higher risk of interruptions if treatment toxicity emerges early, but potential for lower risk of interruptions due to shortened overall treatment duration.	Lower risk due to gradual dose escalation, but potential for higher risk of interruptions due to longer overall treatment duration.
Dose Conformity	Highly conformal with IMRT/VMAT delivery.	Also conformal, particularly in adaptive boost phase.
Logistical Efficiency	Potential for fewer fractions; one upfront plan but may need re-planning for anatomic changes.	Potential for longer treatment schedule; 2–3 upfront plans but often less re-planning required as only boost volumes are treated at the end of treatment when anatomic changes are most apparent.
Clinical Outcomes	Comparable to SEQ in overall survival and tumor control across studies	Comparable to SIB in overall survival and tumor control
Elective Nodal Volume Treatment Timing	Treated concurrently with high-risk volume. Elective nodal volumes treated for the duration of the course	Potential to avoid treatment of low-risk elective volume during the boost portion of treatment to high-risk volume, allowing for faster healing of normal tissues
Dose Per Fraction to Elective Nodal Volume	Lower dose per fraction to elective compared to GTV, but uncertainty about effectiveness if dose per fraction is too low	Allows for homogeneous dose delivery to elective volume and GTV
Dose Per Fraction to GTV	Allows GTV to receive higher dose per fraction (>2 Gy/fx) than the elective nodal volumes (1.5–1.8 Gy/fx)	Homogeneous dose distribution with the same dose per fraction over target volumes (2 Gy/fx)

Source: original creation of the study team.

**Table 2:** Summary of head and neck cancer dose de-escalation trials’ fractionation schemas.

Study	Boost Technique	Elective Dose	BED3	BED10
Yom et al. [26] (NRG-HN005)	SIB-standard SIB-reduced	52.5 Gy/35 fx 48 Gy/30 fx	78.75 Gy 73.6 Gy	60.38 Gy 55.68 Gy
van den Bosch et al. [25] (UP-GRADE-RT)	SIB-control SIB-reduced	50 Gy/34 fx 43 Gy/34 fx	76.5 Gy 61.1 Gy	60.38 Gy 48.4 Gy
Mierwa et al. [27] (Michigan PET trial)	SIB-standard SIB-reduced	56 Gy/35 fx 43.2 Gy/27 fx	85.87 Gy 66.24 Gy	64.96 Gy 50.11 Gy
Sher et al. [23] (UTSW)	SEQ	40 Gy/20 fx	66.7 Gy	48.0 Gy
Maguire et al. [22] (Coastal)	SEQ	36 Gy/18 fx	60.0 Gy	43.2 Gy
Brizel et al. [28] (Duke PET Trial)	SEQ-standard SEQ-reduced	44 Gy/22 fx 36 Gy/18 fx	73.33 Gy 60.0 Gy	52.80 Gy 43.2 Gy
Lee et al. [29] (F-MISO)	SEQ	30 Gy/15 fx	50.0 Gy	36.0 Gy
Tsai et al. [21] (MSKCC)	SEQ	30 Gy/15 fx	50.0 Gy	36.0 Gy
Hanna et al. [30] (ReACT)	SEQ	30 Gy/15 fx	50.0 Gy	36.0 Gy

Source: original creation of study team.

## Data Availability

No new data were created or analyzed in this review. Figure 1 was created to illustrates some of the dosimetric data differences between SEQ and SIB plans in a case of oropharyngeal cancer. Table 1 was created to highlight key differences in SEQ vs. SIB plans, and Table 2 was generated to show the differences in biological dose between standard and de-escalated radiation scheme for both SEQ and SIB plans.
